# Pan-Cancer Analysis Reveals Common and Specific Relationships between Intragenic miRNAs and Their Host Genes

**DOI:** 10.3390/biomedicines9091263

**Published:** 2021-09-18

**Authors:** Baohong Liu, Yu Shyr, Qi Liu

**Affiliations:** 1 Key Laboratory of Veterinary Parasitology of Gansu Province, State Key Laboratory of Veterinary Etiological Biology, Lanzhou Veterinary Research Institute, Chinese Academy of Agricultural Sciences, Lanzhou 730046, China; 2 Center for Quantitative Sciences, Vanderbilt University Medical Center, Nashville, TN 37232, USA; yu.shyr@vanderbilt.edu; 3 Department of Biostatistics, Vanderbilt University Medical Center, Nashville, TN 37232, USA

**Keywords:** miRNAs, host genes, coexpression

## Abstract

MicroRNAs (miRNAs) are small endogenous non-coding RNAs that play important roles in regulating gene expression. Most miRNAs are located within or close to genes (host). miRNAs and their host genes have either coordinated or independent transcription. We performed a comprehensive investigation on co-transcriptional patterns of miRNAs and host genes based on 4707 patients across 21 cancer types. We found that only 11.6% of miRNA-host pairs were co-transcribed consistently and strongly across cancer types. Most miRNA-host pairs showed a strong coexpression only in some specific cancer types, demonstrating a high heterogenous pattern. For two particular types of intergenic miRNAs, readthrough and divergent miRNAs, readthrough miRNAs showed higher coexpression with their host genes than divergent ones. miRNAs located within non-coding genes had tighter co-transcription with their hosts than those located within protein-coding genes, especially exonic and junction miRNAs. A few precursor miRNAs changed their dominate form between 5′ and 3′ strands in different cancer types, including miR-486, miR-99b, let-7e, miR-125a, let-7g, miR-339, miR-26a, miR-16, and miR-218, whereas only two miRNAs with multiple host genes switched their co-transcriptional partner in different cancer types (miR-219a-1 with *SLC39A7/HSD17B8* and miR-3615 with *RAB37/SLC9A3R1*). miRNAs generated from distinct precursors (such as miR-125b from miR-125b-1 or miR-125b-2) were more likely to have cancer-dependent main contributors. miRNAs and hosts were less co-expressed in KIRC than other cancer types, possibly due to its frequent VHL mutations. Our findings shed new light on miRNA biogenesis and cancer diagnosis and treatments.

## 1. Introduction

MicroRNAs (miRNAs) are a class of noncoding small RNAs that negatively regulate the translation and stability of mRNAs. They bind mRNA transcripts in a sequence-specific mode, resulting in degradation or translational repression of corresponding genes [1,2]. miRNAs play pivotal roles in a wide range of biological processes including cell development and differentiation, DNA damage repair, cell death, and intercellular communication [3,4,5]. Around half of miRNAs are located within protein-coding or non-coding genes. These embedded miRNAs are called intragenic miRNAs, and the genes are termed their hosts. Previous studies have reported contradictory results on co-transcriptional patterns between miRNAs and their host genes. While some studies found that intragenic miRNAs were mainly transcribed in parallel with their host transcripts [6], other studies revealed that the majority were transcribed independently through their own promoters [7,8]. The interplay between intragenic miRNAs and host genes makes co-transcription patterns even more complicated [9]. Some intragenic miRNAs target their host genes directly to affect their stability, forming feedback loops that uncouple the coexpression [10,11]. Bioinformatics analysis indicated that approximately 20% of intronic miRNAs suppress their host transcripts [10,11,12]. Moreover, intronic miRNAs play a synergistic or antagonistic role as a partner or enemy to their host genes by targeting the same biological pathways or even the same genes/proteins, which would enhance or decouple the coregulation [13,14,15]. Besides intragenic miRNAs, some intergenic miRNAs have been reported to be co-transcribed with their neighboring genes by readthrough or divergent transcription [16,17]. Since contradictory findings on the relationship of miRNA and hosts were made in small data sets or specific conditions, a systematic and large-scale investigation on their association would facilitate a deeper understanding of miRNA biogenesis and function.

miRNA dysregulation has dramatic consequences on tumorigenesis [18]. They function as tumor suppressors and/or oncogenes to modulate cell proliferation, epithelial-mesenchymal transition, and tumor invasion and metastasis [9,19,20,21,22,23,24]. Our increasing knowledge has boosted the possibilities of miRNA application as potential cancer biomarkers and therapeutic targets. Studies on the transcriptional association of miRNAs and host genes, however, are very limited in cancer. There are only a few studies focusing on specific cancer types or a group of miRNAs [14,25,26,27]. The Cancer Genome Atlas (TCGA) program has generated multi-omics measurements across thousands of tumors, which provides an unprecedented opportunity to study cancer common and specific co-transcriptional patterns between miRNAs and host genes. Dissecting the relationship between miRNAs and host genes in cancer can provide important information for cancer therapy [28,29], can help avoid the unintentional miRNA ablation [30], and assist in miRNA target prediction [31].

## 2. Materials and Methods

### 2.1. mRNA and miRNA Sequencing Datasets

The level III of RNA and miRNA sequencing data for 21 cancer types were downloaded from The Cancer Genome Atlas (TCGA). Samples with both mRNA-seq and miRNA-seq datasets available were kept for downstream analysis. The number of matched samples in each cancer type are listed in Table 1.

### 2.2. miRNA Classification

The genomic coordinates of human miRNAs were extracted from miRBase and release 21.miRNAs were mapped to genomic regions of RNA reference sequences RefSeq Genes (GRCh38/hg38) using in-house Perl scripts. The RefSeq genomic coordinates were downloaded from University of California Santa Cruz (UCSC) genome browser. MiRNAs were called intragenic miRNAs if they were located within the same strand with genes, and otherwise intergenic miRNAs. Based on their relative location to genes, intragenic miRNAs were further classified as exonic, junction, and intronic [32]. If miRNAs could not be assigned into one specific category, they were classified as “mixed”. In addition, we considered two special cases of intergenic miRNAs, which are potentially co-transcribed with their neighboring genes. One is readthrough, where the transcription of one gene goes beyond the normal transcriptional termination site into intergenic regions. miRNAs located in the immediate (<4000 bp) downstream sense region of genes were defined as readthrough miRNAs. The other is divergent, where two polymerase transcribing on both sense and antisense directions from the same promoter. miRNAs located head-to-head of genes (<2000 bp) were defined as divergent miRNAs [16].

### 2.3. Analysis of Transcriptional Association between miRNAs and Host Genes across Cancer Types

Pearson correlation coefficients were used to assess transcriptional association between miRNAs and their host genes in each cancer type using R v.4.1.0 (R Core Team).. A random effects meta-analysis model was used to pool correlations from all cancers. The effect size in each cancer, measured as Pearson correlation coefficients, and the number of samples were combined through random effects meta-analysis to generate an overall effect size and *p*-value. *p*-values were adjusted by Benjamini and Hochberg false discovery controlling method [33]. Meta-analysis was performed using a ‘meta’ package v.4.18-2 (Freiburg im Breisgau, Germany) in R [34].

DGCA v.1.0.1 (New York, NY, USA) was used to perform differential coexpression analysis of each individual miRNA-host pair between cancer types, which computed empirical *p*-values via permutation testing [35]. The differential coexpression analysis was carried out between the two cancer types where the miRNA-host pair obtained the maximum and the minimum correlation coefficients. *p*-values were adjusted by Benjamini and Hochberg false discovery controlling method. Differential correlation analysis was implemented using the DGCA R package [35].

## 3. Results

### 3.1. Human miRNA Classification

The chromosomal coordinates of 1881 miRNAs were obtained from miRBase v21 [32] and were mapped into genes annotated in the Refseq Human Genome annotations hg38. We classified 1881 miRNAs into eight categories based on their genomic locations, intronic, exonic, junction, readthrough, divergent, mixed, antisense, and intergenic (Table 2). A total of 918 miRNAs (48.9%) were embedded within intronic regions of coding or non-coding genes, while 74 (3.9%) and 45 (2.4%) reside in exonic and junction regions of genes, respectively. Recent studies revealed that a small percentage of intergenic miRNAs were potentially co-transcribed with their neighboring genes through two mechanisms. One mechanism is called readthrough, where the transcription of a gene continues beyond the normal transcription termination site into intergenic regions. We found 59 miRNAs (3.1%) located in the immediate (<4000 bp) downstream region and the same strand of genes, which are likely to be transcribed by readthrough transcription. The other is called divergent transcription, defined as two polymerases transcribing on both sense and antisense directions from the same promoter. We discovered 50 miRNAs (2.6%) located on the opposite strand and close to transcriptional start site of genes, which are likely to be transcribed by divergent transcription. In this study, we called these two particular types of intergenic miRNAs (readthrough or divergent) ‘intragenic’ since they potentially share promoters with their neighboring genes. The neighboring genes that miRNAs are potentially co-transcribed with were called ‘host’ genes as well. There were 90 miRNAs (4.8%), which could not be assigned into one specific category. For example, one miRNA is located within the intronic region of one transcript, but the exonic region of another transcript. In this case, the miRNA was assigned into a “mixed” category. In addition, 198 miRNAs (10.5%) located in antisense regions of genes and 447 intergenic miRNAs (23.8%), were excluded from the analysis.

### 3.2. Cancer-Common Transcriptional Relationships between miRNAs and Host Genes

**Figure 1:**
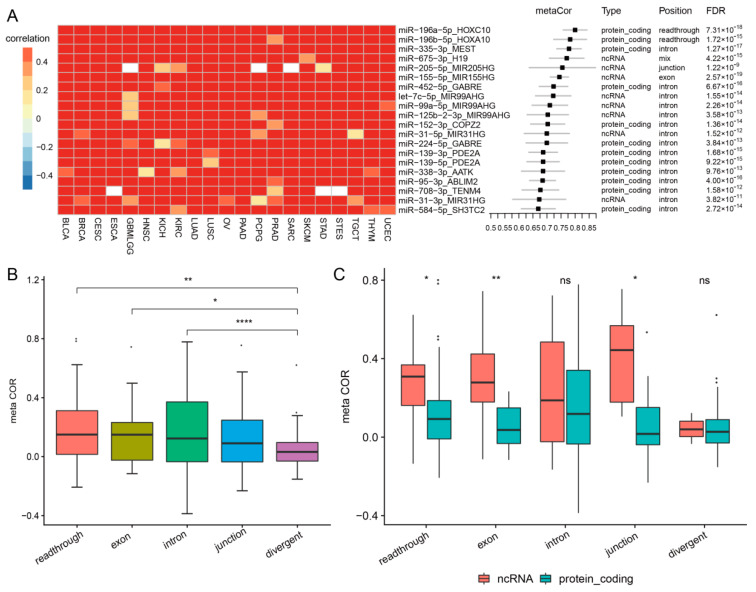
Meta analysis result of all cancers. (**A**). The top 20 coexpressed miRNA-host pairs. (**B**). Meta-correlation on miRNA-host pairs broken into subtypes, readthrough, exonic, intronic, junction and divergent. (**C**). Meta-correlation on miRNA-host pairs broken into protein-coding and noncoding genes. * *p* < 0.05, ** *p* < 0.01, **** *p* < 0.0001.

We performed a meta-analysis on the expression correlation between miRNAs and their hosts from 21 cancer types using a random-effects model. Of 2040 miRNA-host pairs (Supplementary Table S1), 683 can be detected in at least one cancer type (Supplementary Table S2). 79 miRNA-host pairs (11.6%) were found to be consistently and tightly co-expressed across all cancer types (r_meta_ > 0.5 and FDR < 0.01) (Supplementary Table S3), suggesting they share promoters and undergo co-transcription. The top 20 miRNA-host pairs were shown in Figure 1. The most coregulated pairs were miR-196a-5p/HOXC10 (r_meta_ = 0.8 [0.76–0.84], FDR = 7.3 × 10^−18^) and miR-196b-5p/HOXA10 (r_meta_ = 0.78 [0.72–0.84], FDR = 1.7 × 10^−^^15^). miR-196a-5p is generated from two precursors, miR-196a-1 and miR-196a-2. miR-196a-2 is located downstream of HOXC10. The tight coexpression between miR-196a-5p and HOXC10 indicates that miR-196a-2 is the major source of miR-196a-5p expression and it is co-transcribed with HOXC10 by transcriptional readthrough. Similarly, miR-196b-5p is located downstream of HOXA10 and is coexpressed with HOXA10 by transcriptional readthrough. Among the 79 miRNAs, there were 65 intronic, one divergent, four junction, one exonic, five readthrough, and three mixed miRNAs (Supplementary Table S3). miR-1247 is located head-to-head with the DIO3 gene. The high co-transcription between miR-1247-5p and DIO3 across all cancer types suggests that they share the same promoter by divergent transcription (r_meta_ = 0.62 [0.55–0.69], FDR = 7.4 × 10^−^^13^). Of the four junction miRNAs, three are located within noncoding genes (miR-205-5p/MIR205HG, miR-424-3p/MIR503HG, and miR-424-5p/MIR503HG), and one is embedded in a protein-coding gene (miR-1287-5p/PYROXD2). Only one exonic miRNA, miR-155-5p, was co-transcribed with MIR155HG across all cancer types (r_meta_ = 0.74 [0.71–0.78], FDR = 2.6 × 10^−^^19^). Besides two readthrough miRNAs—miR-196a-5p and miR-196b—there are three additional readthrough miRNAs that were also co-transcribed with their upstream genes, including miR-450b-5p and miR-542-3p with MIR503HG, and miR-615-3p with HOXC6. miR-615-3p is not only located in the downstream of HOXC6, but is also embedded in the intronic region of HOXC5. Its tight transcription with HOXC6 instead of HOXC5 suggest its potential regulation mechanism. The three miRNAs belonging to a mixed category, miR-199a-3p, miR-199-5p and miR-675-3p, were all co-transcribed with non-coding genes, DNM3OS and H19 (Supplementary Table S3). DNM3OS and H19 have multiple isoforms, in which miRNAs are located in an exonic region in one isoform or the intronic region. The strong coexpression between miRNAs and their hosts across cancer types suggests that one isoform might dominate the expression.

**Figure 2:**
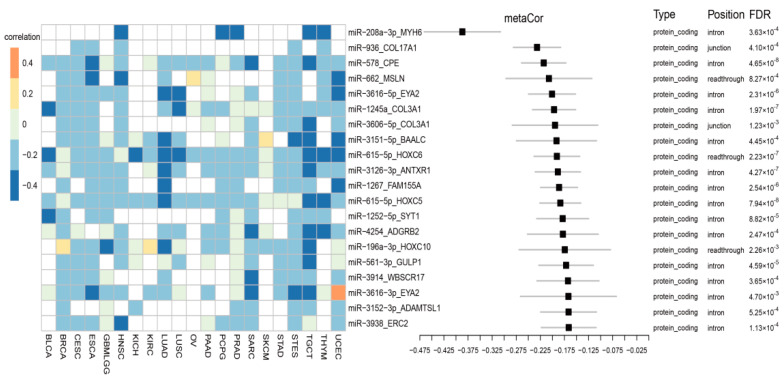
The top 20 negatively coexpressed miRNA-host pairs from a meta-analysis result of all cancers.

Interestingly, 71 miRNA-host pairs (10.4%) were found to be uncorrelated or even weakly inversely correlated in their expressions across all cancer types (r_meta_ < 0 and FDR < 0.01) (Supplementary Table S2), suggesting they might have independent promoters or even competed for promoter usage. The most negatively correlated 20 miRNA-host pairs are shown in Figure 2. For example, expression of miR-208a, an intronic miRNA, was negatively correlated with expression of its host MYH6 in HNSC (r = −0.3, FDR < 0.01), PCPG (r = −0.47, FDR < 0.01), PRAD (r = −0.36, FDR < 0.01), TCGT (r = −0.41, FDR < 0.01), THYM (r = −0.41, FDR < 0.01), and were undetected in other cancer types. The meta-analysis obtained an overall correlation coefficient of −0.39 [−0.46, −0.3] and FDR = 0.0004 (Figure 2). Among the 71 miRNAs, there were 52 intronic, three divergent, five junction, three exonic, and eight readthrough miRNAs (Supplementary Table S2). Compared to 79 miRNAs that were consistently coexpressed with host genes, the 71 miRNAs had a slightly decrease in the proportion of intronic miRNAs (82% vs. 73%), but an increase in divergent, junction, exonic and readthrough ones.

Among 683 pairs, 106 are related to non-coding genes, whereas 577 are involved in protein-coding genes. Although only 1–2% of the human genomes produce proteins, most miRNAs reside in protein-coding genes. The reason for this is probably due to miRNA origin and evolution. Previous studies found that it is a suggestive selective advantage favoring the genomic structure of miRNAs emedding in protein-coding genes to benefit from host genes’ expression control [36,37,38]. There are 495 intronic, 35 exonic, 26 junction, 64 readthrough, 38 divergent, and 25 mixed miRNAs. Comparing different transcriptional categories other than the mixed, we found that divergent miRNAs showed a significantly lower coexpression with their host genes than intronic (*p* = 4.3 × 10^−^^5^), exonic (*p* = 0.02) and readthrough (*p* = 0.006) miRNAs (Figure 1B). Among 38 divergent miRNA-host pairs, only miR-1247 and DIO3 showed strong coexpression across all cancer types (r_meta_ = 0.62 [0.55–0.69], FDR = 7.4 × 10^−^^13^) (Supplementary Figure S1). This suggests that divergent miRNAs are less likely to share promoters with their hosts or more cancer-specific types than other transcriptional categories. In contrast, readthrough miRNAs showed similar co-expression with their hosts as intronic ones. Among 64 readthrough miRNAs, 17 miRNA-hosts had meta-correlation ≥ 0.3 and FDR < 0.01 (Supplementary Figure S2) (Supplementary Table S2), including the widely studied miR-21. miR-21 is located immediately downstream of the vacuole membrane protein-1 (VMP1) gene. The transcription of VMP1 was reported to bypass the polyadenylation signals to include miR-21, thus providing a novel and independently regulated source of miR-21, termed VMP1–miR-21 [39]. Previous studies have reported that exonic miRNAs and host genes were likely to be inversely correlated due to the competition between Drosha processing and canonical splicing [40,41]. Our results, however, found that exonic miRNAs showed a similar expression correlation with host genes as intronic miRNAs. A further investigation found that the high correlation was driven by those exonic miRNAs located in non-coding genes. Nearly half of exonic miRNAs are located in non-coding genes, such as let-7a/MIRLET7BHG, miR-155/MIR155HG, miR22-MIR22HG, miR-600/MIR600HG, and miR-214/DNM3OS. The exonic miRNAs in non-coding genes are more likely to share promoters with their host than those in coding genes (*p* = 0.002) (Figure 1C). Similarly, readthrough and junction miRNAs located in non-coding genes showed a higher coexpression with their hosts than those in coding genes (*p* = 0.03, 0.02, respectively) (Figure 1C). Investigating transcriptional association in each cancer type, exonic/junction miRNAs located in non-coding genes had a higher coexpression than those in coding genes in most cancer types, especially in CESC, PCPG, SARC, TGCT, and THYM (Supplementary Figure S3). In contrast, intronic and divergent miRNAs located in non-coding genes showed a similar coexpression to their hosts with those in coding genes (Figure 1C).

### 3.3. Cancer-Specific Co-Transcription between miRNAs and Host Genes

**Figure 3:**
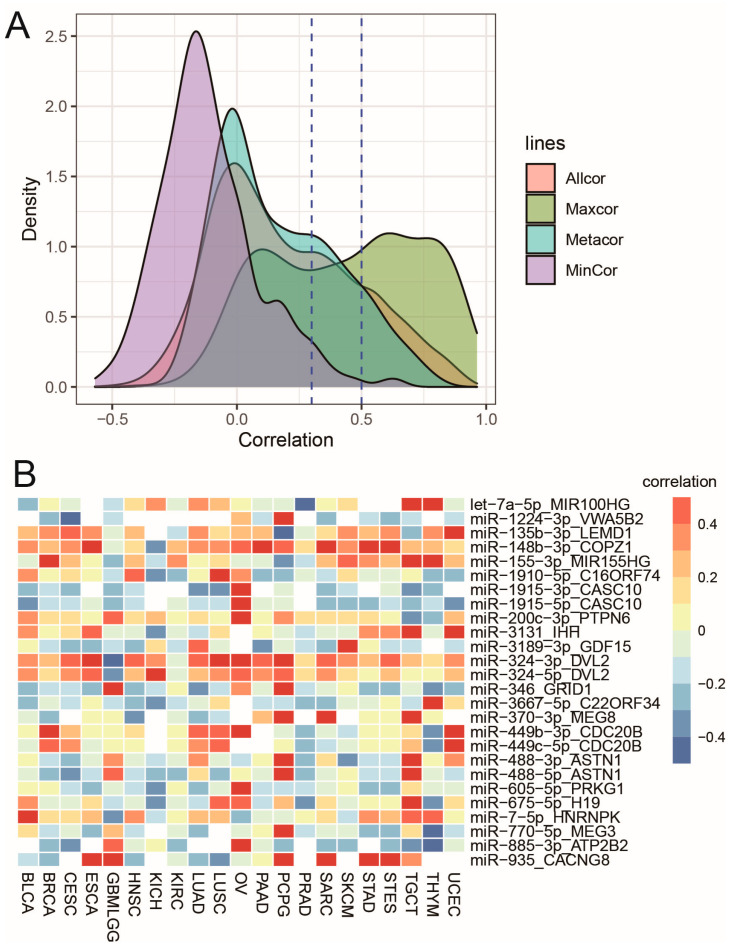
Cancer-specific co-transcriptional patterns between miRNA and host genes. (**A**). The distribution of expression correlations between miRNAs and their hosts. (**B**). The miRNA-host pairs whose maximum correlation >0.5 and minimum correlation <−0.3 across cancer types.

There was a total of 14,343 combinations (683 miRNAs in 21 cancer types); although 8582 showed a positive correlation, only 4224 combinations (29.4%) showed a moderate correlation (r ≥ 0.3 and FDR < 0.01), and 2117 combinations (14.8%) had a strong correlation (r ≥ 0.5 and FDR < 0.01) (Figure 3A). To examine the span of cancer types in which miRNAs were co-transcribed with their host genes, we calculated their maximum and minimum correlations across 21 cancer types. We also performed differential coexpression analysis between the two cancer types, obtaining maximum and minimum correlations for each individual miRNA-host pair to estimate the significance of the correlation difference using DGCA [35]. If the maximum correlation of one miRNA-host pair was greater than 0.5 and FDR < 0.01, they were co-transcribed in at least one cancer type. Although only 79 pairs (11.6%) were consistently and strongly co-expressed across all cancer types (r_meta_ > 0.5 and FDR < 0.01), 324 out of 683 (47.4%) miRNA-host pairs were under tight co-expression in at least one cancer type (rmax > 0.5 and FDR < 0.01) (Figure 3A). Among them, 295 (91%) showed significant coexpression differences between cancer types (z-scores difference > 5 and FDR < 0.01) (Supplementary Table S2). There were 48 miRNA-host pairs (7%) whose maximum correlation coefficients were less than zero. Among the 48 pairs, some miRNAs were not coexpressed with their hosts, suggesting independent promoters, such as miR-3606-5p/COL3A1 and miR-1245a/COL3A1. Some precursor miRNAs were actually transcribed with their hosts, but one miRNA strand was degraded post-transcriptionally. For example, although expressions of miR-450b-3p and its host MIR503HG were uncorrelated (rmax < 0, FDR = 1), pre-miR-450b was actually co-transcribed with MIR503HG through readthrough transcription, which was demonstrated by the tight coexpression of miR-450b-5p with MIR503HG in all cancer types (r_meta_ = 0.52 [0.44–0.62], FDR = 1 × 10^−^^9^). This result indicates that the decoupled correlation between miR-450b-3p and MIR503HG is not due to an independent promoter, but posttranscriptional degradation of miR-450b-3p. Interestingly, we found 26 miRNA-host pairs that were highly coexpressed in some cancer types (r > 0.5 and FDR < 0.01), but were inversely correlated in other cancer types (r < −0.3 and FDR < 0.01) (Figure 3B). They all showed significant coexpression differences across cancer types (z-scores difference > 5 and FDR < 0.01) (Supplementary Table S2). For example, miR-155-3p and MIR155HG were co-transcribed in some cancer types, but were significantly inversely correlated in PCPG (r = −0.31, FDR < 0.01) and uncorrelated in BLCA, ESCA, GBMLGG, KICH, LUAD, LUSC, OV, PAAD, and PRAD (Supplementary Figure S4) (z-scores difference = 9, FDR = 1.3 × 10^−^^20^). Since miR-155-5p and MIR155HG showed a cancer-independent co-transcription pattern, pre-miR-155 is highly likely to transcribe along with MIR155HG. However, post-transcriptional regulation on miR-155-3p in some cancer types are dysregulated, leading to inverse/no correlation. As another example, miR-200c-3p was only strongly coexpressed with its host PTPN6 in OV by readthrough transcription (r = 0.57 and FDR < 0.01), while their expression was uncorrelated in most cancer types and was even inversely correlated in TGCT (Supplementary Figure S5). There was a significant coexpression difference of miR-200c-3p and PTPN6 between OV and TGCT (z-scores difference = 9, FDR = 4.1 × 10^−^^20^). Consistently, previous studies reported that a bypass of the regular PTPN6 polyadenylation signal allows the transcription of the downstream miR-200c [42]. miR-200c was known to be involved in the metastasis and invasion of ovarian carcinoma due to its functional regulation of epithelial-to-mesenchymal transition (EMT) [43,44]. Its co-transcription with PTPN6 provides a potential way to target miR-200c in ovarian cancer.

Our results suggest that transcriptional association between miRNAs and host genes are very heterogeneous across cancer types. While only 79 pairs were consistently co-transcribed in all cancer types, most of them showed a cancer-specific pattern. Although 47.4% of miRNAs shared transcription with their hosts in at least one cancer type, co-expression was decoupled in some cancer types, mainly due to alternative promoter usage, post-transcriptional regulation, or interplay between miRNA and host genes.

### 3.4. Cancer-Specific Selection of Sister miRNA Pairs

**Figure 4:**
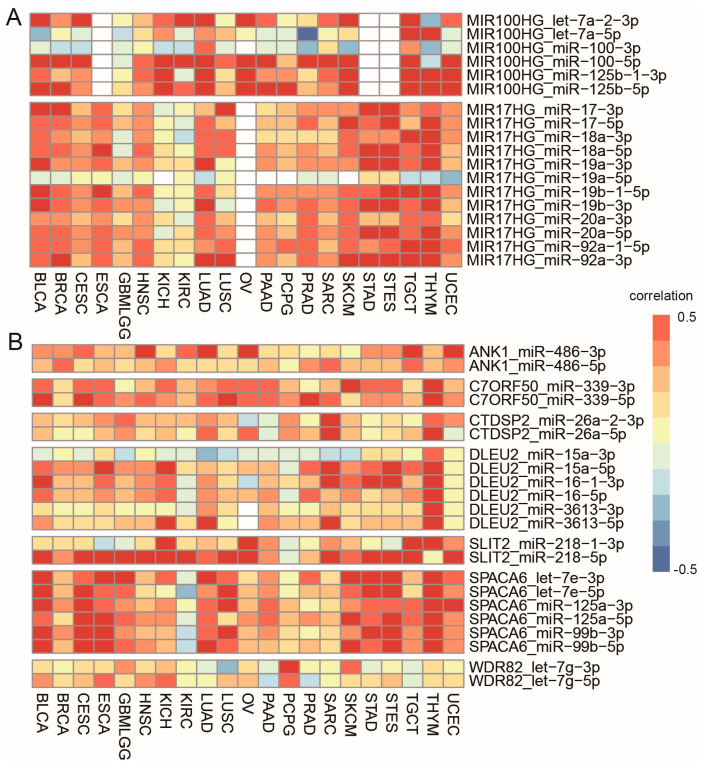
(**A**). miRNA clusters with altered co-transcriptional patterns in different cancer types. (**B**). precursor miRNAs with altered 5′ and 3′ strand that were highly correlated with their host genes in different cancer types.

There are miRNA clusters embedded in genes. We found that miRNAs in the same cluster are either co-transcribed with, or independent from, their host genes simultaneously. We did not observe a co-transcription switch within miRNA clusters across cancer types except miR-100/let-7a-2/miR-125b-1 cluster in MIR100HG and miR-17-92a-1 cluster in MIR17HG. MIR100HG expression was more correlated with pre-miR-100 in KIRC (r = 0.71, FDR < 0.01) with miR-100-5p, r = 0.47, FDR < 0.01 with miR-125b-5p), while it was more correlated with pre-miR-125b in PCPG (r = 0.2 with miR-100-5p, r = 0.66 and FDR < 0.01 with miR-125b-5p) (Figure 4A). Similarly, MIR17HG expression was more correlated with miR-19b in GBMLGG, but with miR-18 in LUSC (Figure 4A).

The study of expression profiles of 5′ and 3′ strands of the same hairpin precursor showed that majority of precursor miRNAs had one dominant and stable form, while the minor form was highly regulated in a cancer-dependent manner. This was reflected in the observation that the dominant form generally had stronger coexpression with its host genes than the minor form in all cancer types when one precursor miRNA was co-transcribed with its host genes. Among the 480 hairpin precursors, we only found seven precursor miRNAs that their co-expression with host genes would switch between the 5′ and 3′ strands, including miR-486, miR-99b, let-7e, miR-125a, let-7g, miR-339, miR-26a, miR-16, and miR-218 (Figure 4B). For example, miR-486-5p was more coexpressed with its host ANK1 than its sister pair miR-486-3p in PRAD and SARC, while it was less coexpressed in CESC, HNSC, KIRC, LUAD, OV, and UCEC (Supplementary Figure S6). They were equally coexpressed with their host in BRCA and TGCT. This suggests that miR-486-5p is selected in PRAD and SARC, while 3p or both strands are concurrently chosen in other cancer types. The alteration of the coexpression partner indicates that the dominant form of a few pre-miRNAs would switch between 5′ and 3′ in some cancer types.

### 3.5. Cancer-Specific Selection of Host Genes and the Main Contributors

**Figure 5:**
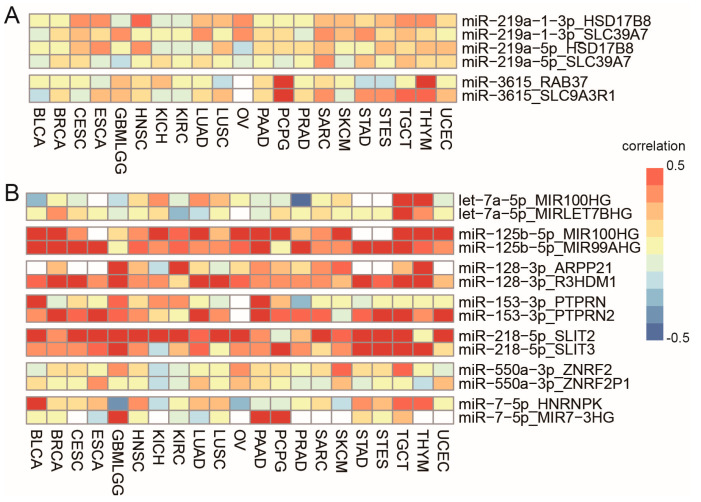
(**A**). miRNAs with altered host genes in different cancer types. (**B**). miRNAs with altered main contributors in different cancer types.

Some miRNAs are located close to more than one gene, meaning they have multiple host genes. For example, miR-3615 is located in the exon region of SLC9A3R1, and also downstream of RAB37. Among the 683 pairs, there are 24 precursor miRNAs with more than one host gene. Of the 24 precursor miRNAs, only two miRNAs switched their hosts in different cancer types, miR-219a-1 and miR-3615 (Figure 5A). miR-219a-1 is located downstream of both HSD17B8 and SLC39A7. It was co-transcribed with HSD17B8 in CESC, ESCA, HNSC, and THYM (r > 0.3, FDR < 0.01), while it was coexpressed with SLC39A7 in GBMLGG, SARC and STAD (r > 0.3, FDR < 0.01) (Supplementary Figure S7). miR-3615 is located in the exon region of SLC9A3R1, and also downstream of RAB37. It was co-transcribed with RAB37 in THYM (r > 0.5, FDR < 0.01), while it was coexpressed with SLC9A3R1 in SARC, STAD, STES, and TGCT (r > 0.3, FDR < 0.01) (Supplementary Figure S8). The switch in coexpressed partners suggests alternative promoter usage in different cancer types.

Some miRNAs are generated from multiple precursors, labeled with -1 and -2. Among the 683 pairs detected in our study, there are 15 miRNAs derived from multiple precursors. Of the 15 miRNAs, seven miRNAs switched their main contributors in different cancer types, adding one more layer of transcriptional regulation, including let-7a-5p, miR-125b-5p, miR-128-3p, miR-153-3p, miR-218-5p, miR-550a-3p and miR-7-5p (Figure 5B). For example, miR-125b-5p are generated from two precursors, miR-125b-1 or miR-125b-2, which are located in intronic regions of MIR100HG and MIR99AHG, separately. miR-125b-5p was strongly coexpressed with MIR100HG in SKCM (r = 0.86, FDR < 0.01), but with MIR99AHG in ESCA, STAD, and STES (r > 0.5, FDR < 0.01) (Supplementary Figure S9).

### 3.6. Comparison of Co-Transcription Patterns across Cancer Types

**Figure 6:**
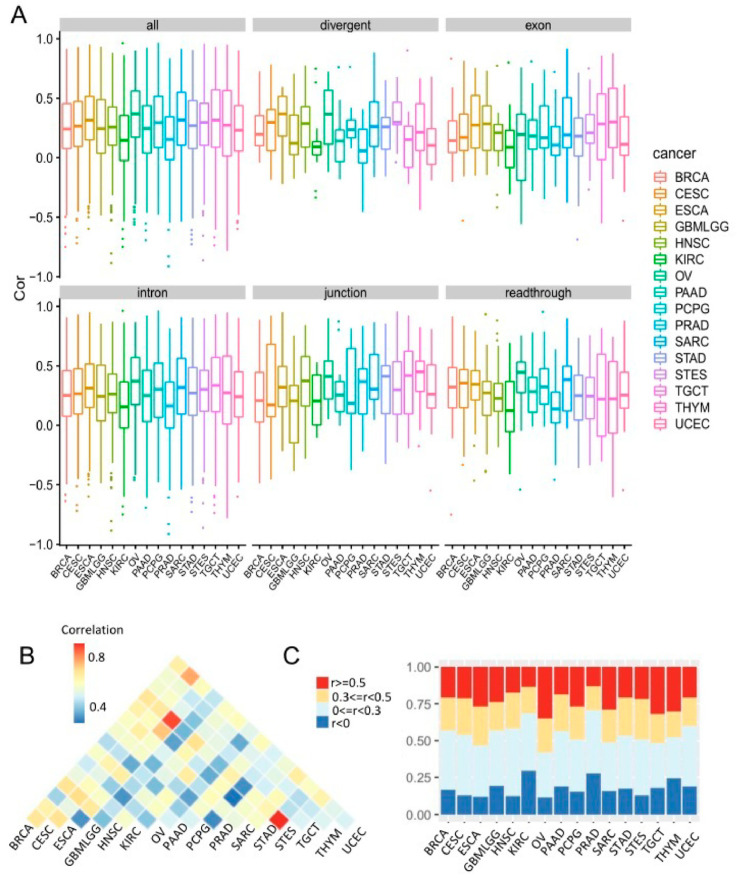
(**A**). Expression correlation between miRNAs and hosts across cancer types. (**B**) Correlation similarity between cancer types. (**C**). The distribution of expression correlation in each cancer type.

To compare co-transcription patterns across cancer types, we randomly sampled 100 patients from each cancer type and computed the correlation coefficients between miRNAs and hosts. We excluded those with less than 100 samples and kept 16 cancer types. Among the 16 cancer types, KIRC showed the lowest coexpression of miRNA-host pairs, while OV had the highest coexpression (Figure 6A). The difference was even dramatic in the transcriptional categories of readthrough and junction. Modur et al. reported that there is a large proportion of cancers that have widespread defects in mRNA transcription elongation, especially in kidney renal clear cell carcinoma [45]. Cancers with transcription elongation defects display spurious transcription and defective mRNA processing of genes characterized by long genomic length; 48% of KIRC patients have mutations in VHL gene in the TCGA dataset. The VHL protein was reported to bind tightly and specifically to subunits of Elongin (SIII), which activates transcription elongation by RNA polymerase II [46]. The low co-expression mutation in KIRC might be partly caused by frequent mutations in VHL.

STAD and STES were most similar in the co-transcriptional association between miRNAs and host genes (r = 0.8, *p* < 0.001), followed by ESCA and STES (r = 0.85, *p* < 0.001). This suggests that co-transcriptional associations are characterized by substantial tissue specificity. In contrast, GBMLGG and OV showed most dissimilar patterns from other cancer types (Figure 6B). We categorized miRNA-host pairs by correlation coefficients into strong (r ≥ 0.5), moderate (0.3 ≤ r < 0.5), weak (0 ≤ r < 0.3), and negative (r < 0). OV has the largest percentage of pairs (36.7%) with a strong correlation, followed by TGCT (31.1%), while KIRC and PRAD showed the smallest percentage of strong pairs (13.8% and 13%, respectively) (Figure 6C).

## 4. Discussion

We performed a comprehensive investigation on transcriptional associations between miRNAs and their host genes across 21 cancer types using TCGA datasets. We detected 79 consistently and strongly coexpressed miRNA-host pairs across all cancer types, where host genes can be used as a proxy for the expression profiles of the embedded miRNAs. This provides a potential way to target miRNAs by affecting gene expression of their hosts. For example, the miR-106b-93-25 cluster was co-transcribed with MCM7 across all cancer types (r_meta_ > 0.5, FDR < 0.01). The miR-106b-93-25 cluster, located in the intronic region of MCM7, is composed of highly conserved miR-106b, miR-93 and miR-25, which are upregulated in multiple cancer types [47,48]. It plays an important role in tumorigenesis, especially as an omco-miR in breast cancer [49]. MCM7, the host gene of the miR-106b-93-25 cluster, and a transcription factor, is also upregulated in many cancers and its high expression is related to poor prognosis [50,51]. Yang et al. reported that the suberoylanilide hydroxamic acid (SAHA, histone deacetylase inhibitors) treatment significantly suppressed expression of the miR-106b-93-25 cluster, as well as its host gene MCM7 in HCC cells. They demonstrated that the transcriptional repression of the miR-106b-93-25 cluster and MCM7 by SAHA was associated with deacetylation of histone H4 but not H3 localized at the MCM7 promoter. They revealed that SAHA repressed the transcription of miR-25, miR-93 and miR-106b by repressing their host genes, MCM7 [52]. Although miR-106b-93 cluster was reported to have an independent primary transcript unit from its host gene [53], our results showed that miR-106b/25 were significantly co-expressed with their host gene MCM7 across all the 21 cancers. This suggests that SAHA may have therapeutic potential for patients with other tumors beyond HCC that overexpress MCM7 and the miR-106b-93-25 cluster.

Our study also revealed an independent transcription between miRNAs and hosts that are commonly observed across cancer types. One reason for independent transcription is that transcriptional regulatory machineries for intragenic miRNAs might be disparate from those of their host genes [8,54]. Previous research reported that over one-third of intronic miRNAs have their own promoters (Polymerase II or III) [8]. For example, miR-1908, located in the first intron of FADS1, was not coexpressed with FADS1 in any cancer types (Supplementary Table S2). Consistently, miR-1908 and FADS1 have been reported to be independently transcribed [55]. The competition/crosstalk between microprocessor cleavage and splicing also leads to uncorrelated expression or even inverse correlation [40]. For example, miR-198 is in the 3′ untranslated region (exon 11) of FSTL1. We found that expression of miR-198 and FSTL1 was unrelated or even inversely correlated in all cancer types (Supplementary Table S2). Previous studies have also reported that an inverse correlation between FSTL1 (pro-migratory) and miR-198 (anti-migratory) demonstrated the regulatory switch in orchestrating wound re-epithelialization [41].

Although some miRNA-host pairs showed either co-transcription or independent transcription across all cancer types, most pairs had a cancer-specific pattern. Their expressions were highly correlated in some cancer types, but the correlation was decoupled in other cancer types. Special attention should be paid for the design and construction of miRNA-to-host gene targeting. For example, miR-504 resides within an intron of the FGF13 gene. FGF13/miR-504 is upregulated in a subset of lung cancer and is a facilitator of cancer progression [56]. The expression of the FGF13/miR-504 is repressed by p53, while miR-504 directly targets p53 mRNA, defining an additional p53-regulatory feedback loop. The coordinated action of FGF13 and miR-504 reinforces the quenching of p53 activity in lung cancer. Although miR-504 was reported to inhibit cell proliferation and promote apoptosis in glioma [57], a low expression of FGF13 and miR-504 both indicate poor survival (Supplementary Figure S10), suggesting that they are functionally synergistic. Our study found the coordinated expression of FGF13 and miR-504 in most cancer types, such as GBMLGG (r = 0.64, FDR < 0.01), LUAD (r = 0.64, FDR < 0.01), LUSC (r = 0.57, FDR < 0.01), PCPG (r = 0.66, FDR < 0.01), SKCM (r = 0.52, FDR < 0.01), TGCT (r = 0.58, FDR < 0.01), and THYM (r = 0.78, FDR < 0.01). However, they were only weakly coexpressed, or even were not coexpressed in some cancer types, such as CESC (r = 0.12, FDR = 0.04), ESCA (r = 0.11, FDR = 0.2), PRAD (r = 0.27, FDR < 0.01), STAD (r = 0.21, FDR < 0.01), and STES (r = 0.17, FDR < 0.01) (Supplementary Figure S11). There was a significant coexpression difference between THYM and ESCA (z-scores difference = 7.8, FDR < 2.6 × 10^−^^14^). Therefore, targeting FGF13 should be examined carefully to determine whether the intentional or unintentional disruption of its intronic miR-504 may confound the effect in specific cancer types. The reason for decoupled coexpression between miRNA and host genes in certain cancer types are complicated, and involve alternative promoters usage, post-transcriptional regulation, and/or interplay between miRNA and host genes. Recent high-throughput advances in experimental data of transcription start sites, histone marks preferring promoters (such as H3K4me3 and DNase I hypersensitivity sites), and miRNA target identification provide an unprecedented opportunity to study cancer-specific promoters and miRNA-gene interactions, which will help untangle the causes.

We found that exon/junction miRNAs located in non-coding genes were more likely to be co-transcribed with their hosts than those embedded in coding genes. miRNAs located in the exon/junction regions of protein coding genes have been reported to have no or even an inverse correlation with their host genes because of the competition between spliceosome and the microprocessor [41,57,58]. For example, miR-198 is embedded in the 3′ untranslated region (exon 11) of FSTL1 and their expression was unrelated, or even inversely correlated in all cancer types (Supplementary Table S2). In contrast, expression of miRNAs located in non-coding genes had a higher co-correlation with their hosts. This is probably due to the reason that noncoding genes serve exclusively to produce miRNAs rather than other functional units, leading to a positive correlation. For example, miR-155 and MIR155HG were strongly co-transcribed across all the 21 cancers. MIR155HG has been known as the primary miRNA of miR-155 and small molecule inhibitors of the MIR155HG/miR-155 axis would be a useful anti-cancer drug [59].

Readthrough and divergent miRNAs are special, whose transcription is a byproduct of their neighboring genes. Divergent miRNA is a product of bi-directional transcription of active promoters. Previous research has identified some bidirectional promoter regions transcribing a miRNA and protein-coding genes simultaneously [16]. Among the 38 divergent miRNA-host pairs, we only found miR-1247, located head to head with DIO3, to be co-transcribed with DIO3 across all the 21 cancer types (r_meta_ = 0.62, FDR = 7.4 × 10^−^^13^). Others were either showed to have a cancer-specific co-transcription pattern, such as miR-34/BTG4, or had independent transcription, such as miR-3188/JUND (Supplementary Table S2). The low likelihood of divergent miRNAs co-transcribed with their hosts is due to easy disruption of bidirectional organization by transposon insertion, recombination, or other genome rearrangement events [60]. Readthrough miRNAs, on the other hand, are generated by a continuous transcription beyond a normal stop sign from an active gene. Readthrough miRNAs were more likely to be co-transcribed with their hosts than divergent miRNAs, and had a similar coexpression with intronic miRNAs. Of the 64 readthrough miRNA-host pairs, five had meta-correlation coefficients greater than 0.5, and 17 greater than 0.3, including the widely studied pair miR-21/VMP1 [61].

miRNAs are deeply involved in tumorigenesis and progression, either acting as tumor promotors or tumor suppressors [62,63,64]. Therefore, targeting oncomiRNAs and mimicking tumor suppressor miRNAs to normalize the gene regulatory work and signaling pathways holds great promise to reverse the phenotype in cancerous cells [65,66]. The comprehensive investigation on co-transcriptional patterns between intragenic miRNAs and host genes in the study not only facilitates the development of better strategies for targeting or mimicking intragenic miRNAs, but also expands our knowledge on the function and network of well-studied host genes and helps improve gene therapy in the cancer treatment. One limitation in our study is the lack of normal tissues. The comparative studies between tumor and matched normal tissues would help identify those important dysregulated miRNA-host pairs related to tumorigenesis.

## Figures and Tables

**Table 1:** Summary of miRNA and mRNA sequencing datasets from 21 different cancers.

Cancer	Description	Paired Samples
BLCA	Bladder urothelial carcinoma	56
BRCA	Breast invasive carcinoma	458
CESC	Cervical and endocervical cancers	304
ESCA	Esophageal carcinoma	183
GBMLGG	Glioma (Brain Lower Grade Glioma)	512
HNSC	Head and Neck squamous cell carcinoma	228
KICH	Kidney Chromophobe	66
KIRC	Kidney renal clear cell carcinoma	204
LUAD	Lung adenocarcinoma	79
LUSC	Lung squamous cell carcinoma	69
OV	Ovarian serous cystadenocarcinoma	288
PAAD	Pancreatic adenocarcinoma	178
PCPG	Pheochromocytoma and Paraganglioma	179
PRAD	Prostate adenocarcinoma	493
SARC	Sarcoma	257
SKCM	Skin Cutaneous Melanoma	97
STAD	Stomach adenocarcinoma	232
STES	Stomach and Esophageal carcinoma	415
TGCT	Testicular Germ Cell Tumors	150
THYM	Thymoma	120
UCEC	Uterine Corpus Endometrial Carcinoma	139

Cancer: Abbreviations for each cancer; Description: Full names for each cancer. Paired samples: Number of paired mRNA and miRNA samples for each cancer.

**Table 2:** miRNA classification.

Type	Number	Percent (%)
Intronic	918	48.9
Exonic	74	3.9
Junction	45	2.4
Readthrough	59	3.1
Divergent	50	2.6
Mixed	90	4.8
Antisense	198	10.5
Intergenic	447	23.8

## Data Availability

The RNA-seq datasets were downloaded from TCGA (http://cancergenome.nih.gov, accessed on 12 October 2017). Other data are available in the paper and the Supplementary File.

## Supplementary Materials

The following are available online at https://www.mdpi.com/article/10.3390/biomedicines9091263/s1, Figure S1: Correlation between readthrough miRNAs and their host genes across 21 cancer types. Figure S2: Correlation between divergent miRNAs and their host genes across 21 cancer types. Figure S3: miRNA-host expression correlations broken into protein coding and non-coding genes in each cancer type. Figure S4: Expression correlation between miR-155-3p and MIR155HG. Figure S5: Expression correlation between miR-200c-3p and PTPN6. Figure S6: Expression correlation between miR-486-5p/3p and ANK1. Figure S7. Expression correlation between miR-219a-1 and HSD17B8/SLC39A7. Figure S8: Expression correlation between miR-3615 and RAB37/SLC9A3R1. Figure S9: Expression correlation between miR-125b-5p and MIR100HG/MIR99AHG. Figure S10: (A) The survival analysis of FGF13 in LGG. (B) The survival analysis of miR-504-5p in LGG. Figure S11: Expression correlation between miR-504 and FGF13. Table S1: Intragenic miRNA-host gene pairs in human. Table S2: Meta analysis results for miRNA-host gene pair correlations across 21 cancers. Table S3: 79 miRNA-host pairs consistently and strongly coexpressed in all cancer types.
