# Supplementary figures and images for: Pan-Cancer Analysis Reveals Common and Specific Relationships between Intragenic miRNAs and Their Host Genes
Source: Biomedicines. 2021 Sep 18;9(9):1263. doi: 10.3390/biomedicines9091263 (PMC8471046; doi:10.3390/biomedicines9091263)

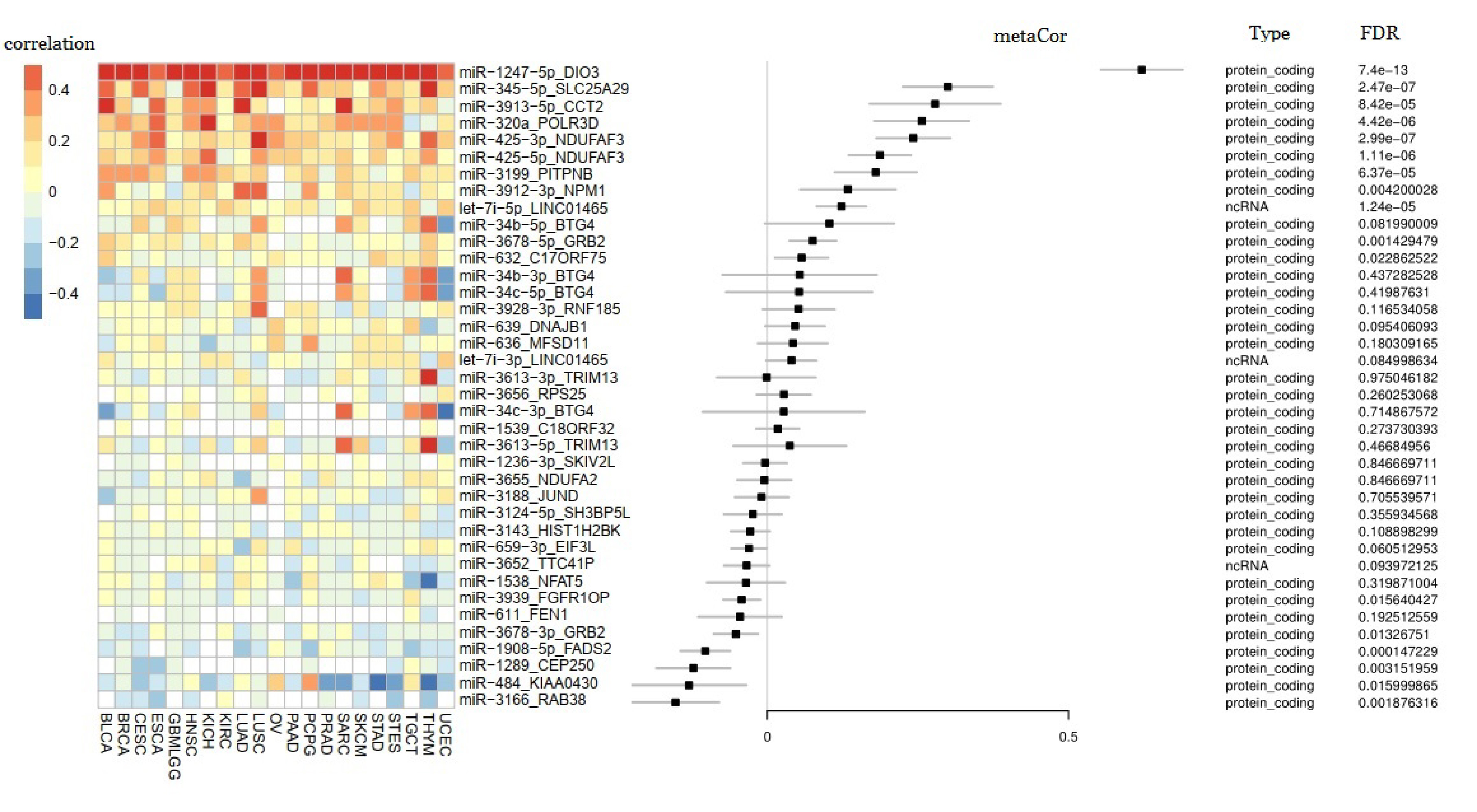

Supplement: Supplementary file 1 [file biomedicines-09-01263-s001.zip › Figure. S1.jpg]

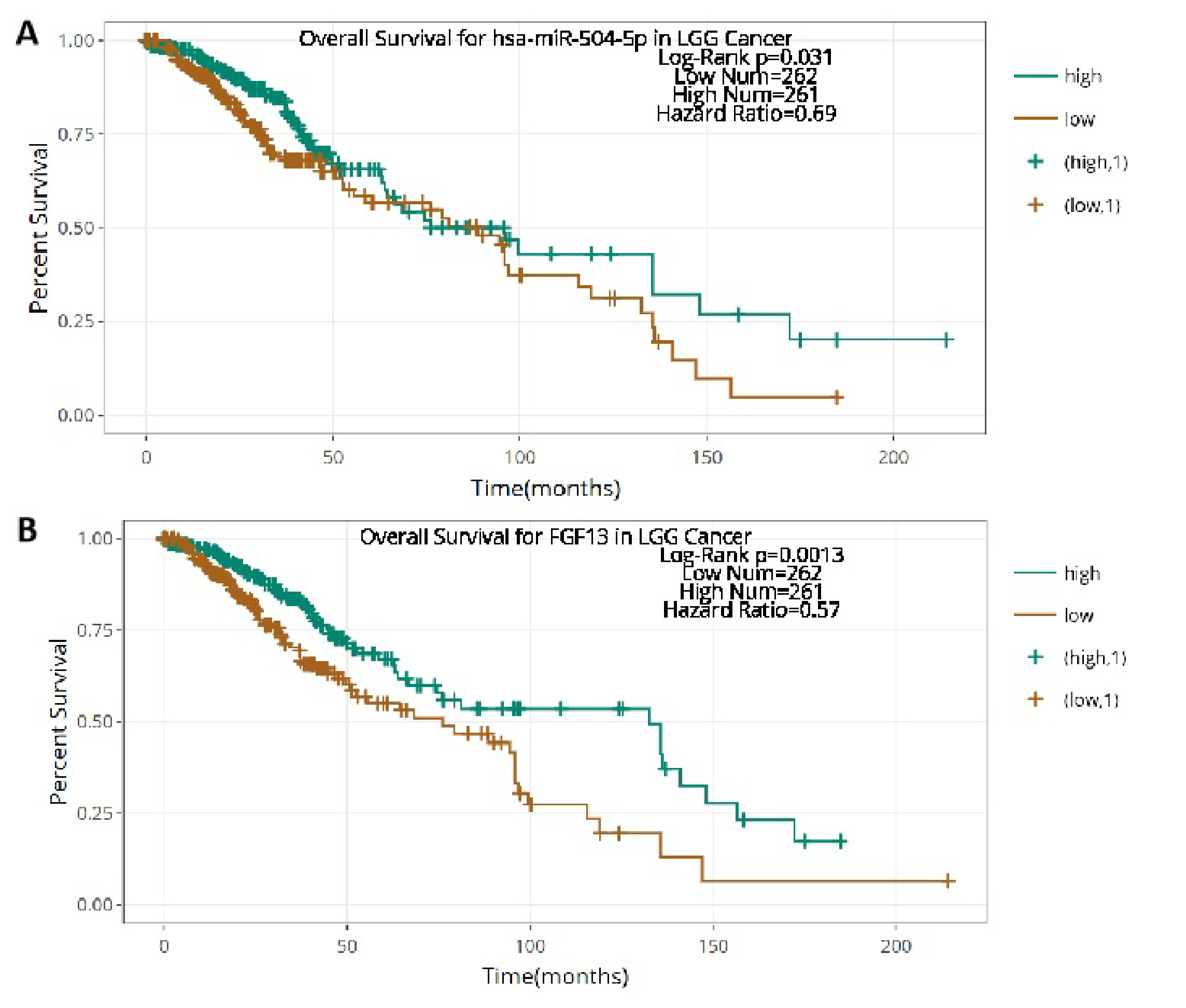

Supplement: Supplementary file 1 [file biomedicines-09-01263-s001.zip › Figure. S10.jpg]

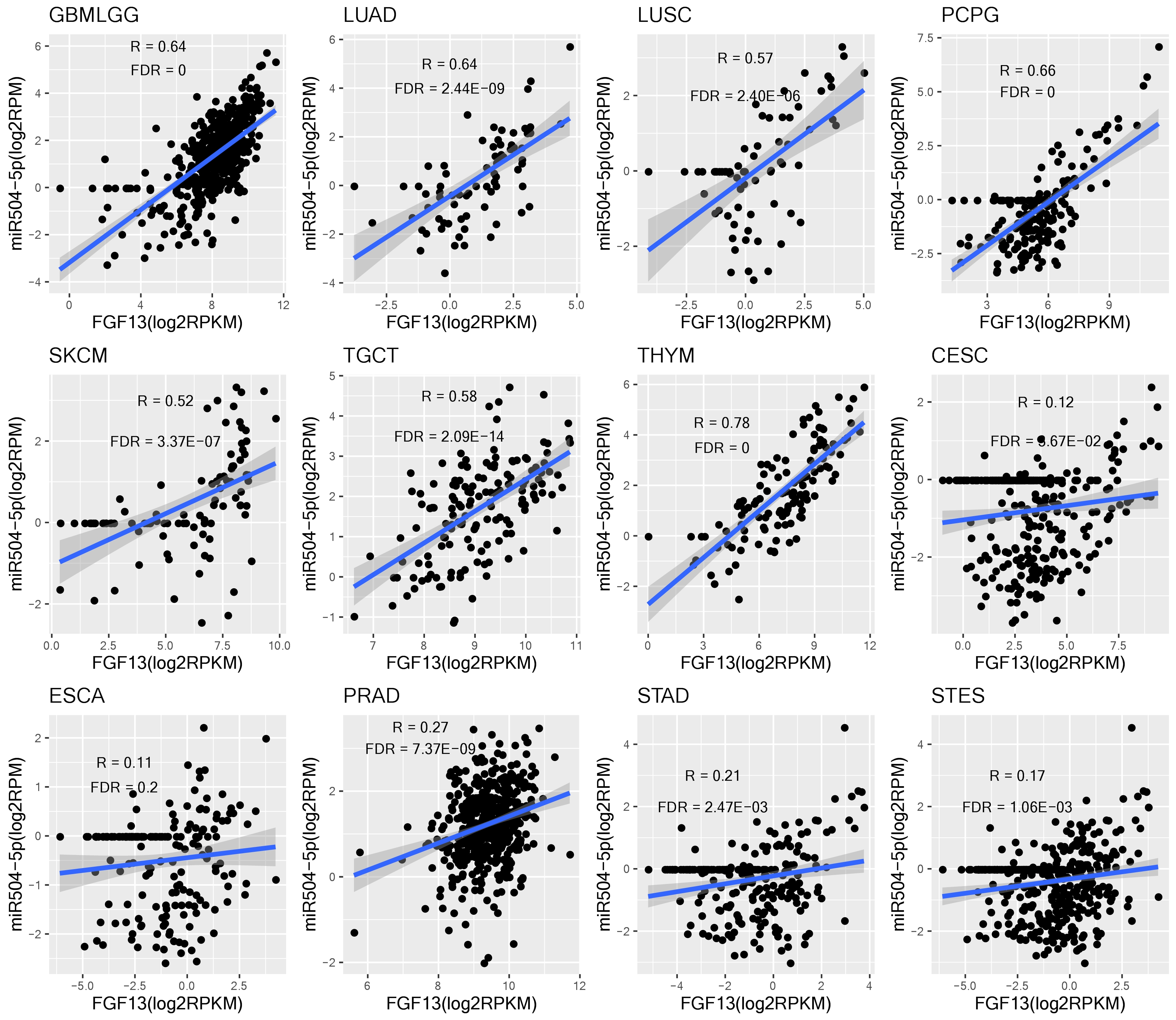

Supplement: Supplementary file 1 [file biomedicines-09-01263-s001.zip › Figure. S11.jpg]

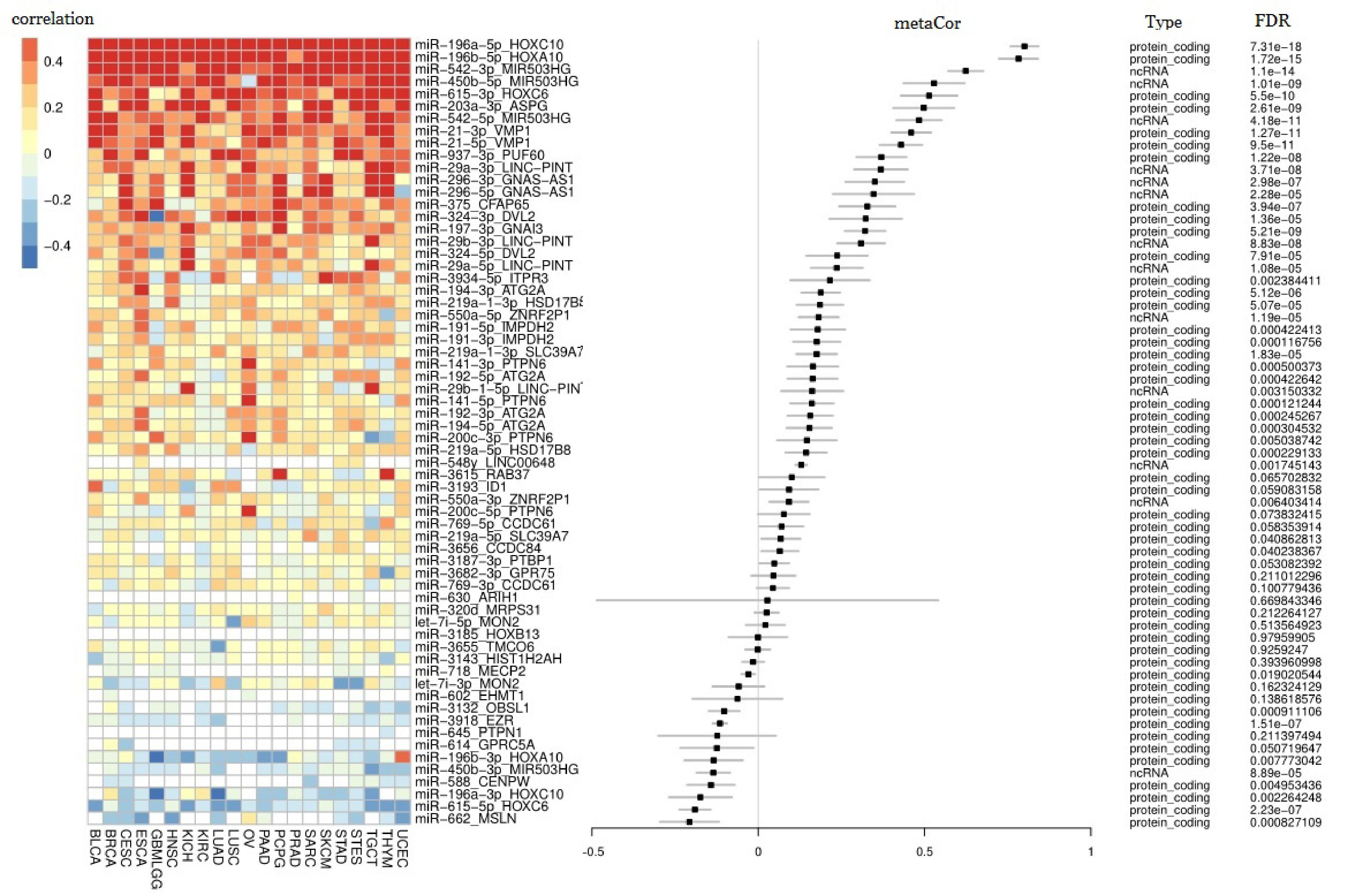

Supplement: Supplementary file 1 [file biomedicines-09-01263-s001.zip › Figure. S2.jpg]

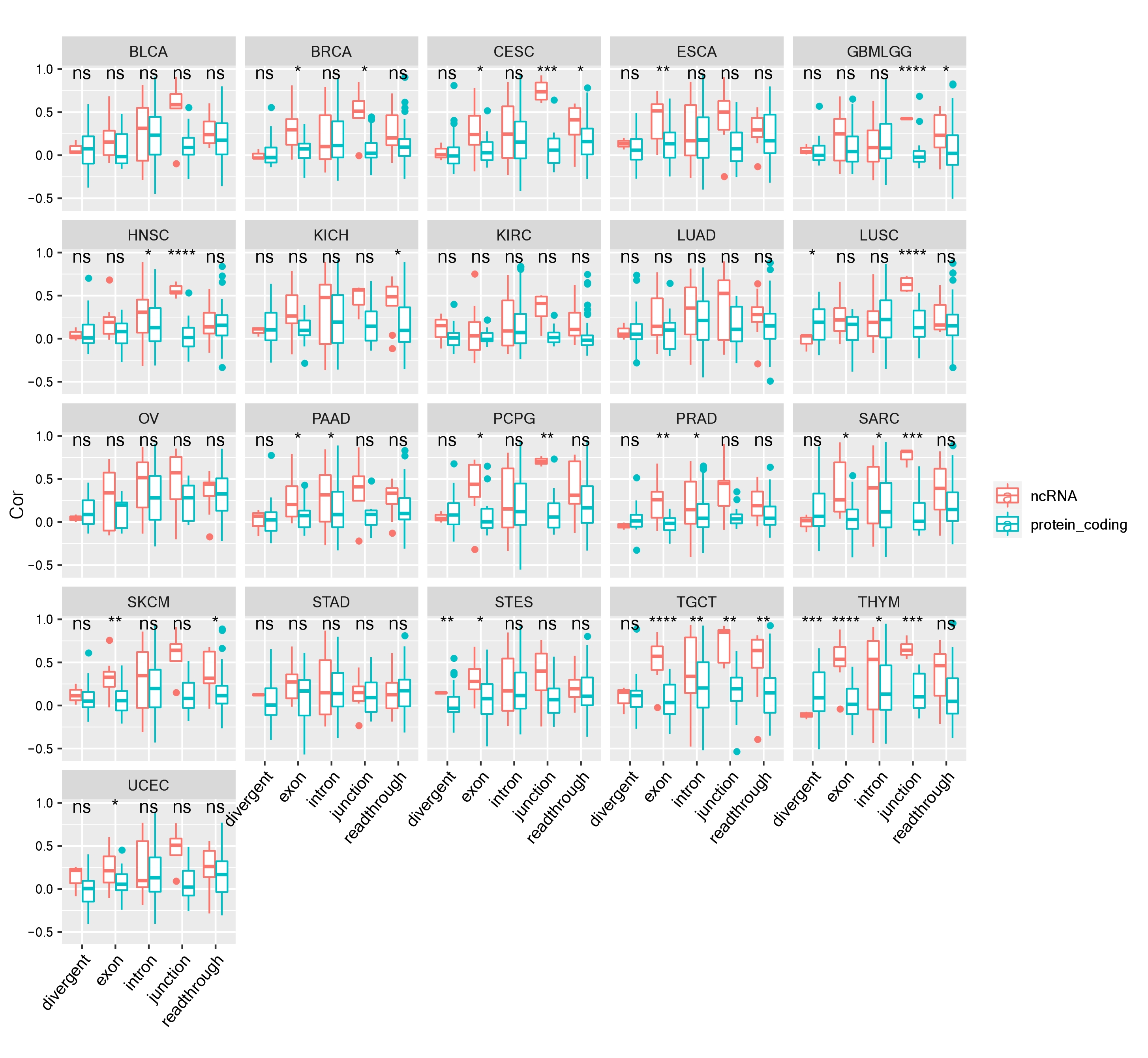

Supplement: Supplementary file 1 [file biomedicines-09-01263-s001.zip › Figure. S3.jpg]

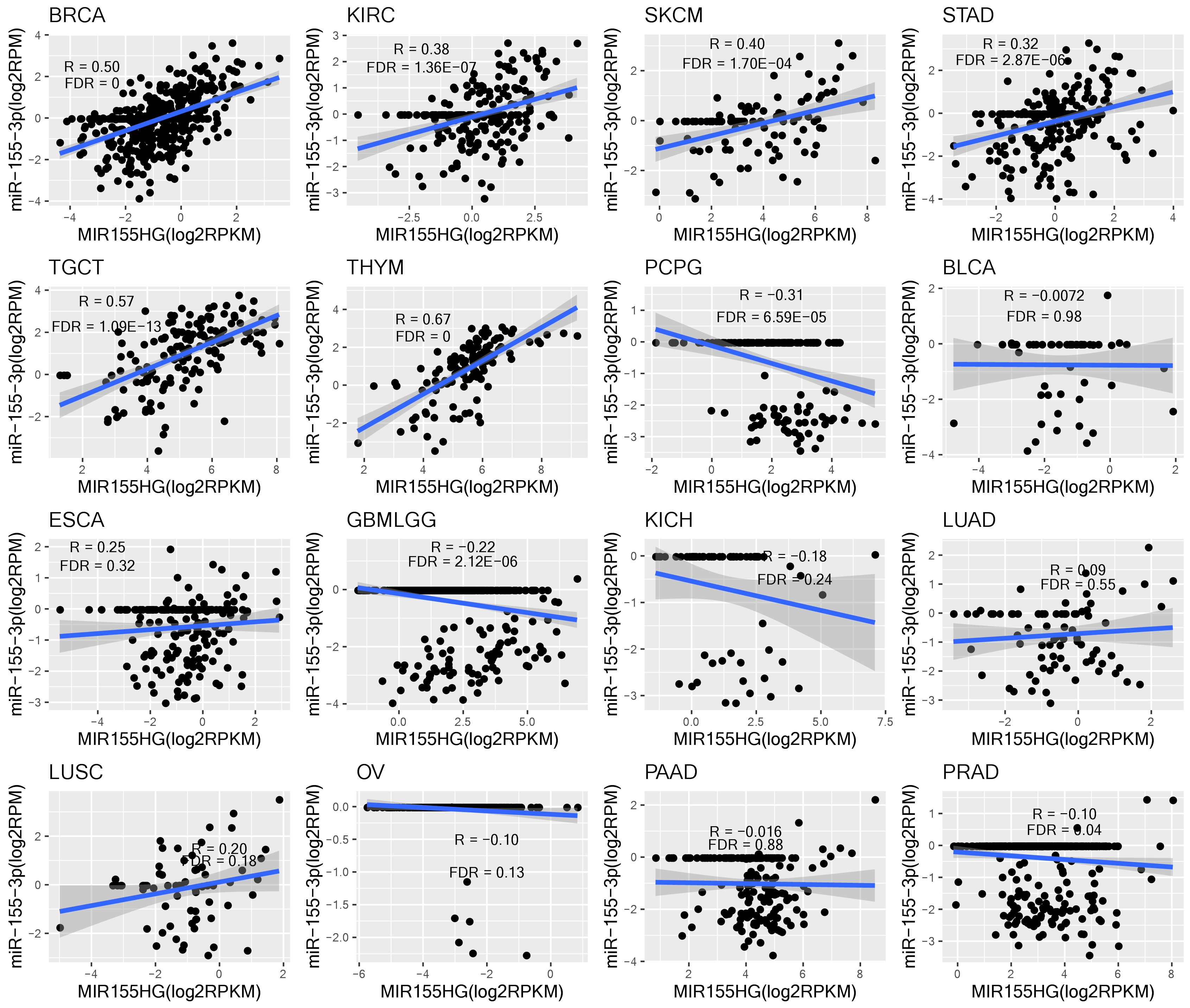

Supplement: Supplementary file 1 [file biomedicines-09-01263-s001.zip › Figure. S4.jpg]

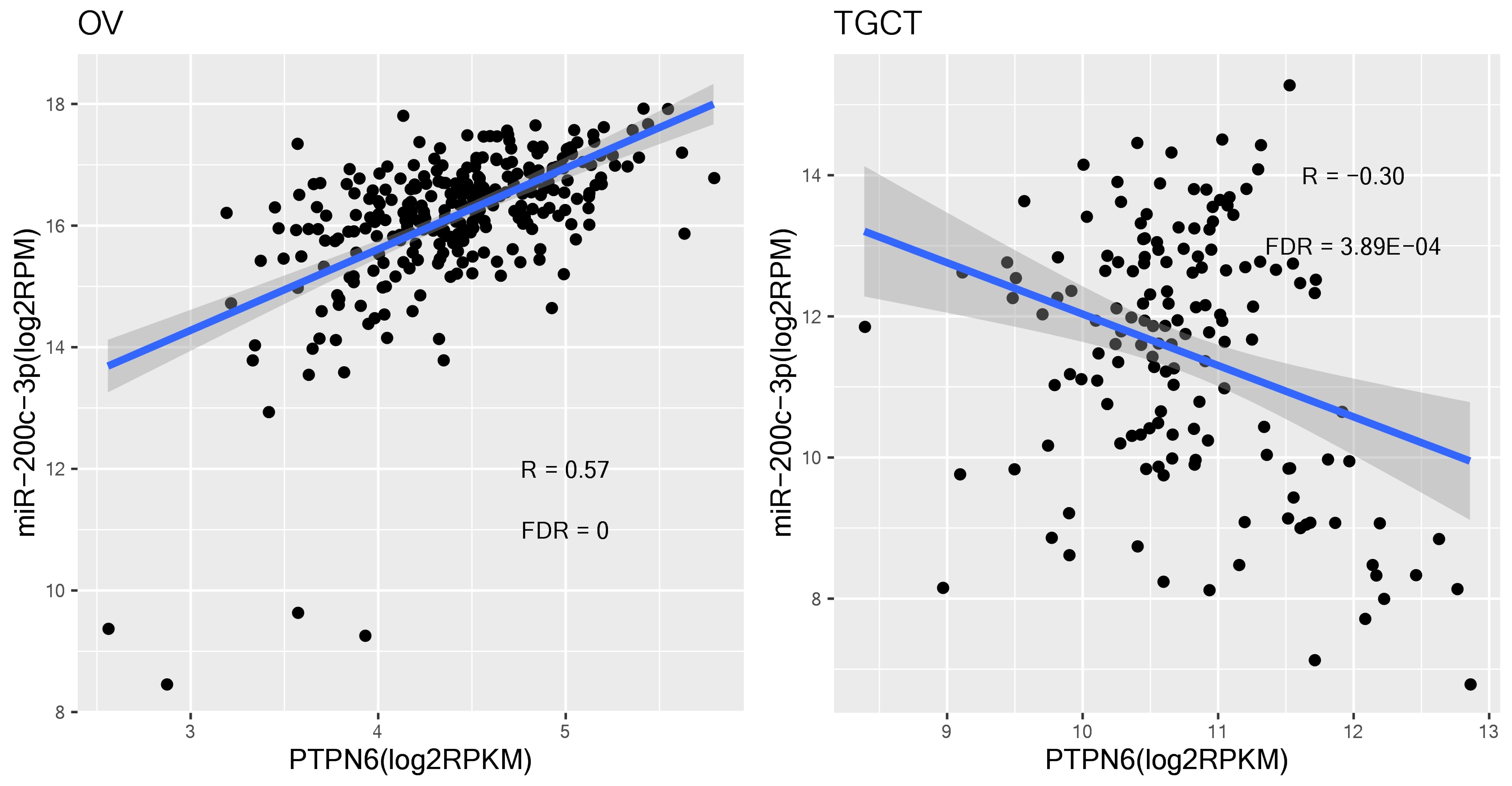

Supplement: Supplementary file 1 [file biomedicines-09-01263-s001.zip › Figure. S5.jpg]

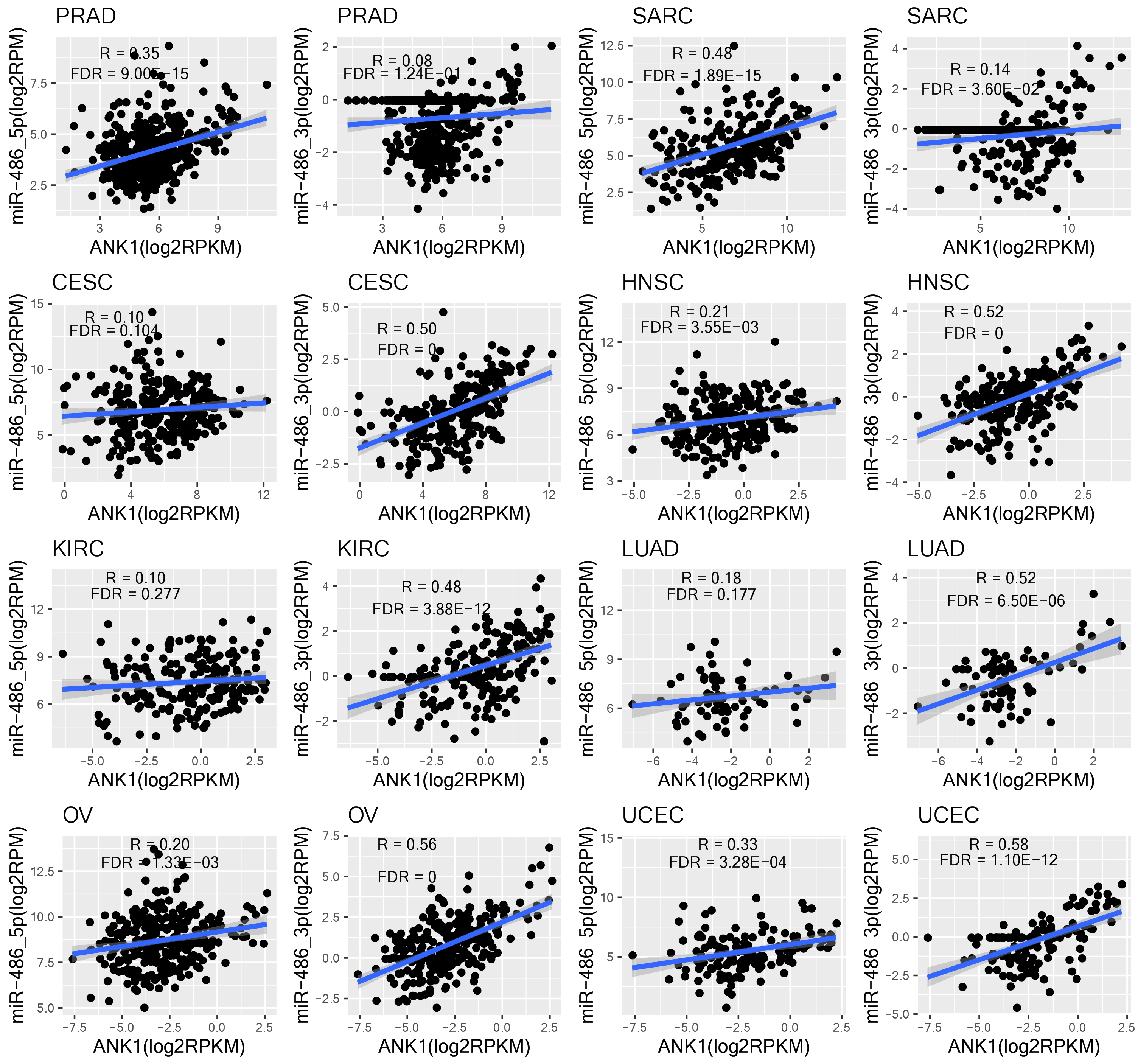

Supplement: Supplementary file 1 [file biomedicines-09-01263-s001.zip › Figure. S6.jpg]

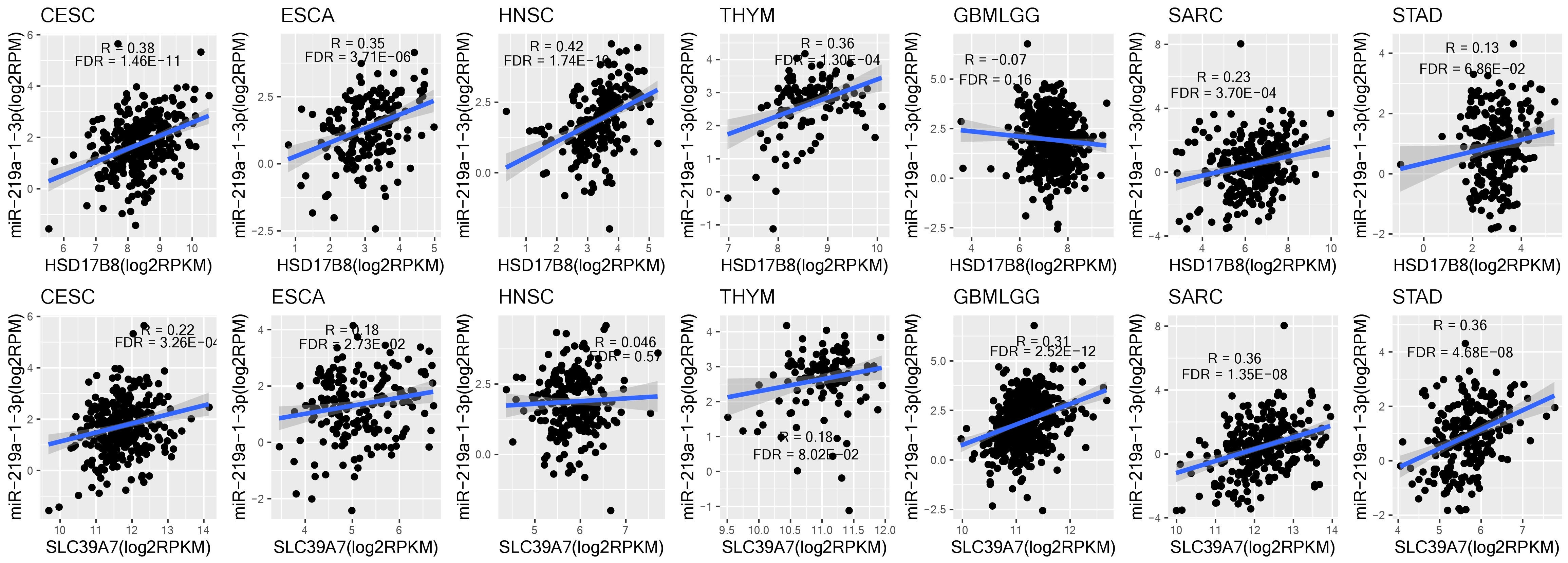

Supplement: Supplementary file 1 [file biomedicines-09-01263-s001.zip › Figure. S7.jpg]

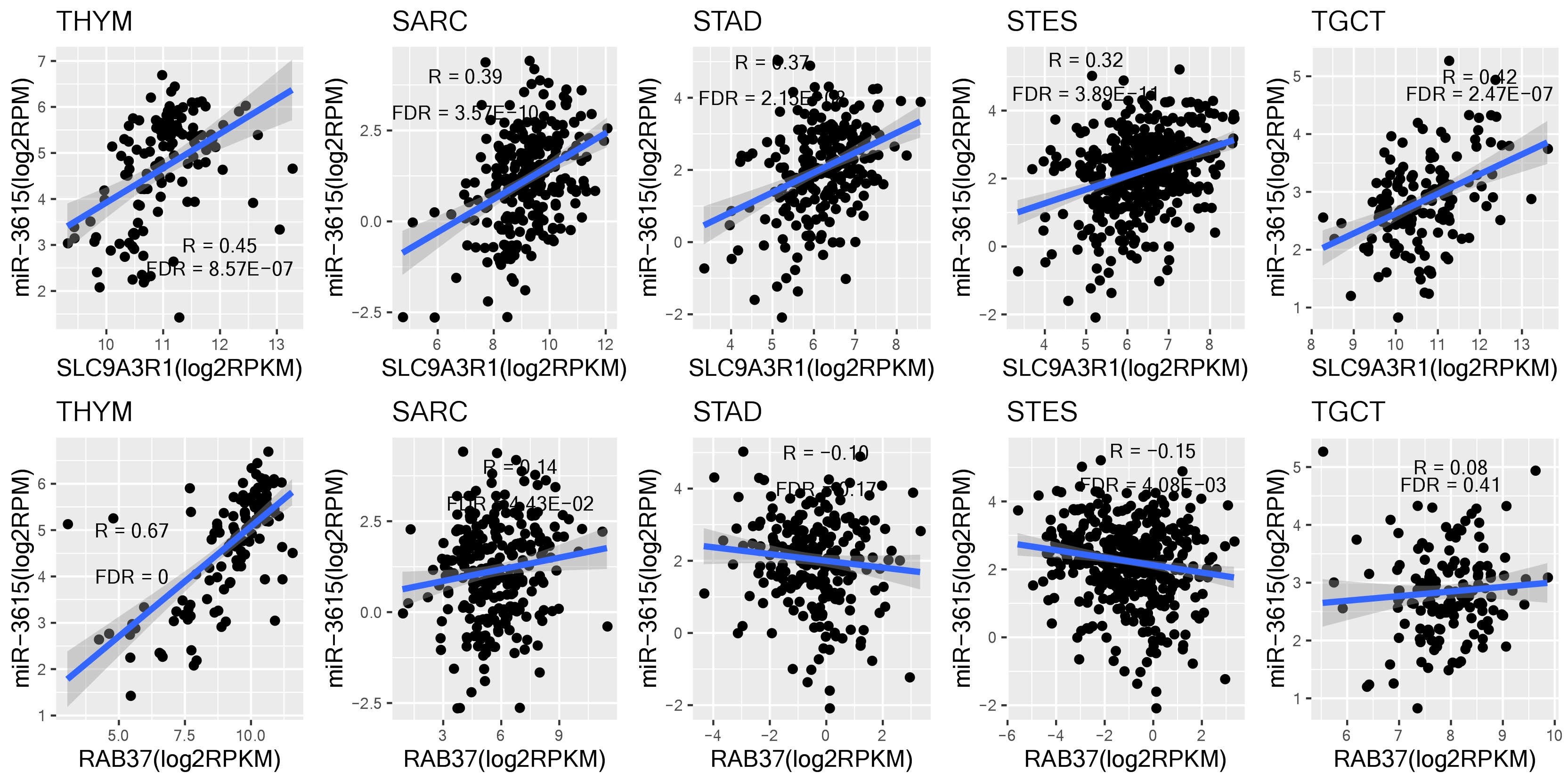

Supplement: Supplementary file 1 [file biomedicines-09-01263-s001.zip › Figure. S8.jpg]

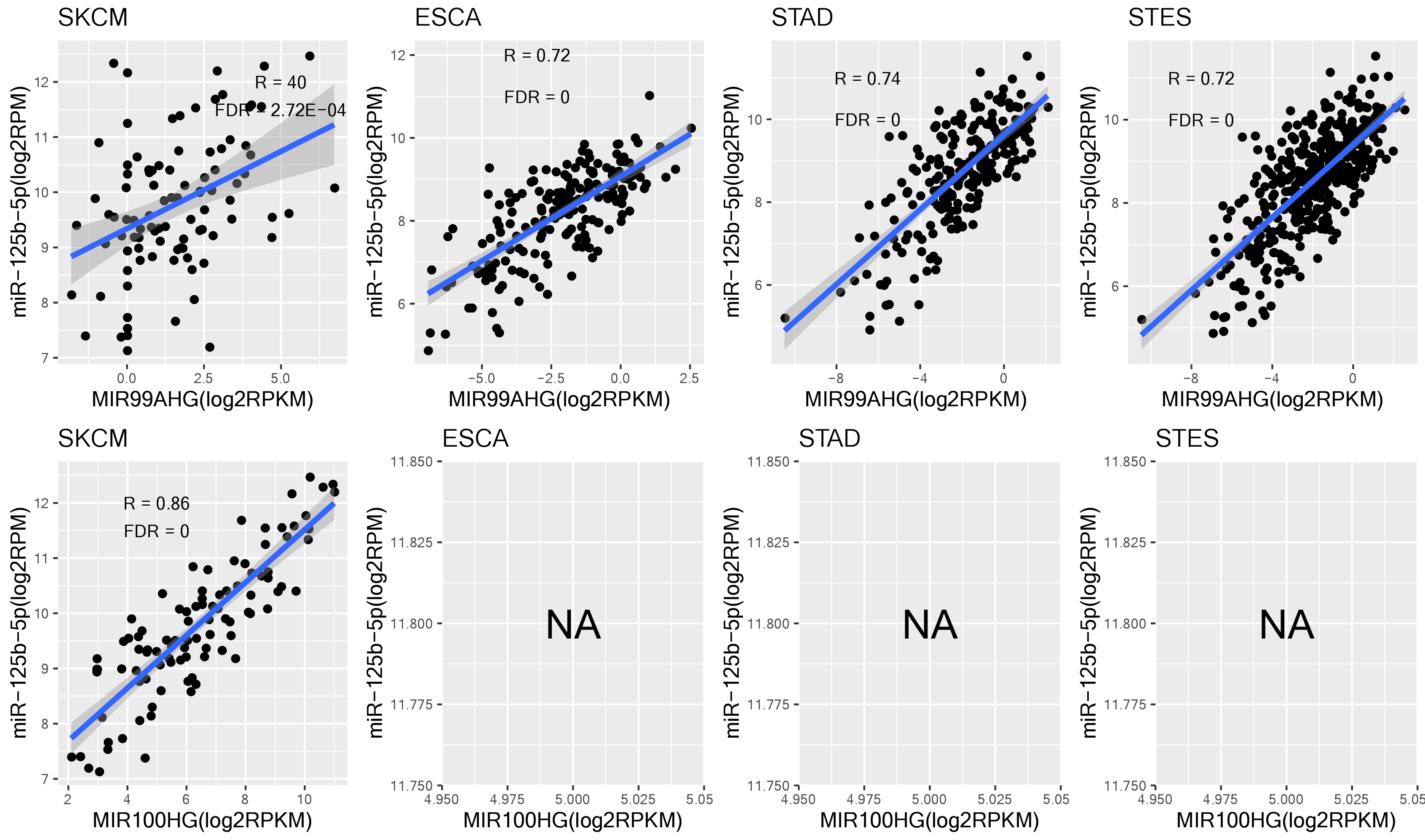

Supplement: Supplementary file 1 [file biomedicines-09-01263-s001.zip › Figure. S9.jpg]
